# Continuous Quality Improvement and Comprehensive Primary Health Care: A Systems Framework to Improve Service Quality and Health Outcomes

**DOI:** 10.3389/fpubh.2018.00076

**Published:** 2018-03-22

**Authors:** Janya McCalman, Ross Bailie, Roxanne Bainbridge, Karen McPhail-Bell, Nikki Percival, Deborah Askew, Ruth Fagan, Komla Tsey

**Affiliations:** ^1^Centre for Indigenous Health Equity research, Central Queensland University, Cairns, Australia; ^2^The Cairns Institute, James Cook University, Townsville, QLD, Australia; ^3^Centre for Research Excellence in Integrated Quality Improvement, University Centre for Rural Health, University of Sydney, Sydney, NSW, Australia; ^4^Poche Centre for Indigenous Health, Sydney Medical School, University of Sydney, Sydney, NSW, Australia; ^5^University of Technology Sydney, Ultimo, NSW, Australia; ^6^The University of Queensland, Brisbane, QLD, Australia; ^7^Inala Indigenous Health Service, Queensland Health, Inala, QLD, Australia; ^8^Gurriny Yealamucka Health Service, Yarrabah, QLD, Australia; ^9^College of Arts, Social Science and Education, James Cook University, Townsville, QLD, Australia

**Keywords:** continuous quality improvement, systems approach, Indigenous, primary health care, integration, social determinants of health

## Abstract

Continuous quality improvement (CQI) processes for improving clinical care and health outcomes have been implemented by primary health-care services, with resultant health-care impacts. But only 10–20% of gain in health outcomes is contributed by health-care services; a much larger share is determined by social and cultural factors. This perspective paper argues that health care and health outcomes can be enhanced through applying CQI as a systems approach to comprehensive primary health care. Referring to the Aboriginal and Torres Strait Islander Australian context as an example, the authors provide a systems framework that includes strategies and conditions to facilitate evidence-based and local decision making by primary health-care services. The framework describes the integration of CQI vertically to improve linkages with governments and community members and horizontally with other sectors to influence the social and cultural determinants of health. Further, government and primary health-care service investment is required to support and extend integration and evaluation of CQI efforts vertically and horizontally.

## Introduction

Continuous quality improvement (CQI) approaches in primary health care have enabled adherence to best practice clinical guidelines and improved regularity of client attendance (1). Implementation of CQI approaches has also resulted in a CQI workforce, appropriate health system supports, and engagement with other organizations and community members (2). Yet, since the relative contribution of health care on health outcomes is estimated to account for only between 10 and 20% of gain (3–5), the improvement of health-care performance alone is not enough to achieve improved health outcomes. We argue that optimal benefit for health care from CQI will be attained through a systems approach, whereby comprehensive primary health-care services are better enabled to make evidence-based and locally responsive decisions through integrating CQI vertically in linkages with governments and community members and horizontally in linkages with other sectors. Further, government and primary health-care service investment is needed to support and extend such integration of CQI efforts.

## What is CQI and What are its Impacts?

Continuous quality improvement in health care is “a structured organizational process for involving people in planning and executing a continuous flow of improvement to provide quality health care that meets or exceeds expectations” (6) (p. 4). O’Neill et al. (7) highlighted four key elements of CQI approaches as follows: (1) implemented in or by a health-care service; (2) collecting qualitative or quantitative data on intervention effectiveness, impacts, or success; (3) reporting client (or caregiver) health outcomes; and (4) aiming to change how delivery of care is routinely structured. CQI models vary according to local diversity between primary health-care services, the CQI team, and the external environment. There is no clear evidence that any one CQI model is better than another (8, 9).

Multiple impacts of implementing CQI in clinical health care are reported internationally. Studies report reduced hospital admissions among patients with chronic conditions and reduced emergency department visits among older patients (10). In addition, studies document increased workforce capabilities, capacities, and enthusiasm to deliver best practice primary care (11, 12). Improved organizational efficiencies also arise from the availability of good quality, timely local data such as a self-sustaining ability to recognize, analyze, and improve quality issues by controlling and allocating available resources more effectively (13, 14). Aggregating the quantified benefits is somewhat challenging because of the diversity of CQI models, variations in implementation, and the methodological challenges of studying such complex interventions (9).

## The Example of Indigenous Australian Primary Health Care: To What Extent CQI has been Implemented?

A Centre of Research Excellence in Integrated Quality Improvement (CRE-IQI) was established in Australia in 2015 to support improved Aboriginal and Torres Strait Islander (hereafter respectfully termed Indigenous) health outcomes by accelerating and strengthening large-scale primary health-care quality improvement efforts (15). The CRE-IQI builds on and extends CQI approaches that have been implemented by Indigenous primary health-care services since 2002 to promote best practice clinical health care. CQI strategies have been used in various forms in Indigenous Australian primary health-care services over many years. Three quality improvement projects have been particularly influential in terms of their wide scope and reach: the Healthy for Life, Audit for Best Practice in Chronic Disease and One21seventy, and Australian Primary Care Collaborative projects (12, 16–18). Additionally, a range of activities have also been implemented at local levels (12).

These projects have partially achieved many of the conditions required to support implementation of CQI across Indigenous Australian primary health-care services (12). First, there is a very strong grassroots interest in clinical CQI in Indigenous health care (12). Second, CQI audit tools and processes have been developed in chronic disease, preventive care, maternal health, child health, mental health, rheumatic heart disease, youth health, sexual health, and child development, and implemented on a broad scale (19, 20). Third, in the Northern Territory, use of CQI audit tools and processes has resulted in increased workforce capabilities, capacities, and enthusiasm to deliver best practice primary health care. Capabilities include a “strong leadership for CQI, participation in CQI of a range of staff at all levels, ability to adapt CQI processes to local contexts, provision of training and technical support to implement CQI [and] availability of high quality and timely data” (11) (p. 73). Fourth, through CQI, data are shared between primary health-care services, policy-makers, and researchers and utilized jointly to analyze variation in quality of care and system factors (19). Largely missing, however, are strong management structures, systems, resourcing, and a culture of CQI to: (1) support CQI across all levels of the health system from active engagement of consumers/patients in primary health-care service decision making to government policy and resourcing that supports data and evidence-based decision-making processes and (2) integration with other sectors to influence the social and cultural determinants of health.

## How can CQI Efforts in Indigenous Australian Primary Health Care be Enhanced?

**Figure 1:**
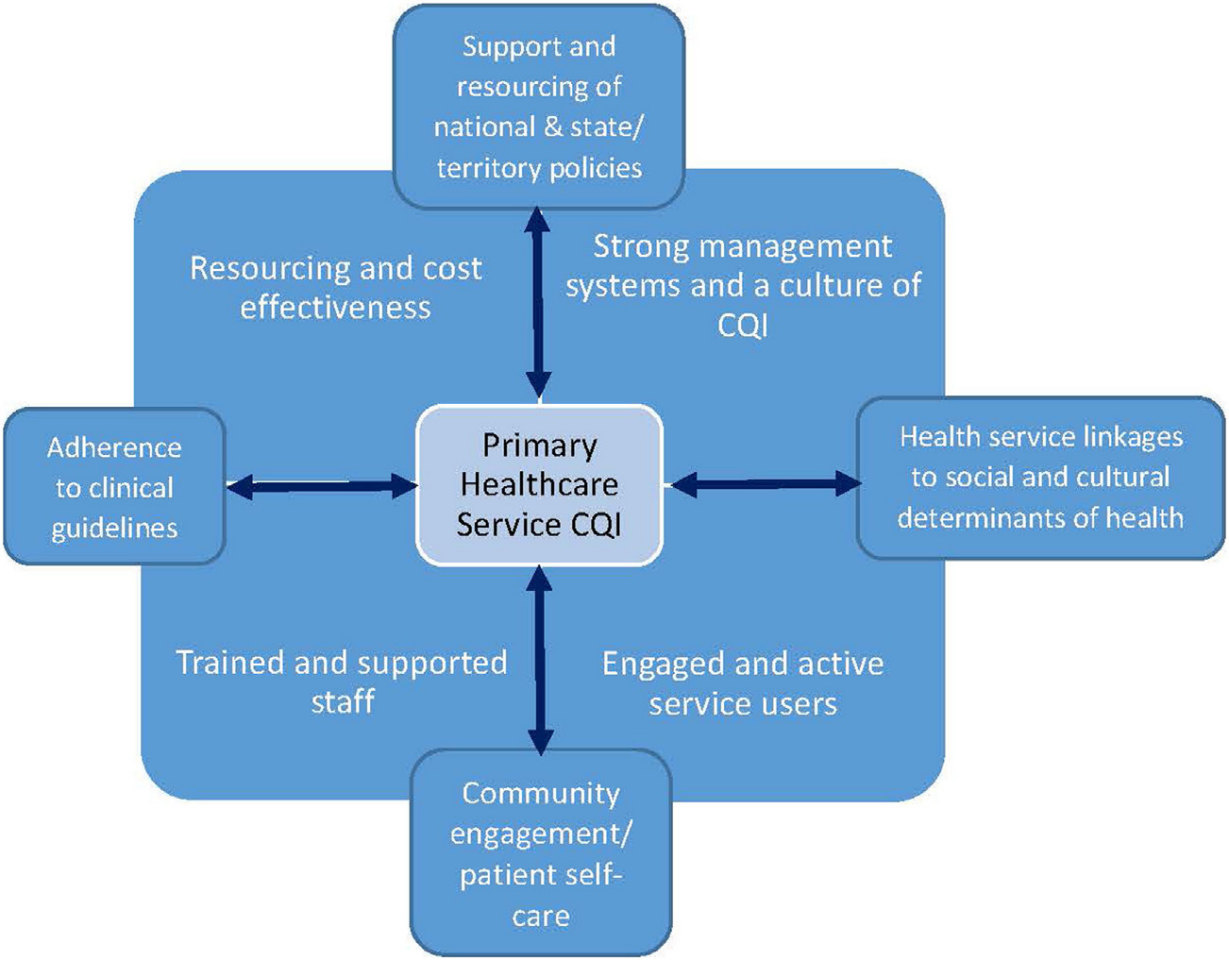
The vertical and horizontal enhancement of continuous quality improvement (CQI).

In May 2017, the authors of this paper and 22 other Indigenous health practitioners, researchers, and CQI facilitators met at the bi-annual meeting of the CRE-IQI in Brisbane. One of the authors (Ross Bailie) described a framework (Figure 1 below) to explicate a systems approach for expanding the scope and strengthening CQI efforts to improve service quality and health outcomes. A systems’ approach to health recognizes that individuals are embedded within social, cultural, political, and economic systems that shape behaviors and access to resources necessary for health (21, 22). Systems approaches can be useful for understanding and clarifying the complex effects of historical, social, and environmental circumstances on Indigenous people’s health across the life course (23). The emphasis of a systems framework lies not in explicating the causal effect of a single factor but in understanding the functioning of the system as a whole and predicting their behaviors so that adaptations can be made to produce desired effects (24).

The systems framework (Figure 1) depicts the potential for primary health-care services to support and extend integration of CQI efforts in two dimensions: vertically across the health system and horizontally across sectors. By vertical integration, we mean the application of CQI across all levels of health systems, from community engagement and patient care (represented at base of diagram) to state, territory, and national policy levels (top of diagram). By horizontal integration, we mean not only the incorporation of CQI into clinical guideline adherence at the individual primary health-care service site (left side of diagram) but also linkages and advocacy for the social and cultural determinants of health (right side of diagram). The social and cultural determinants of health include connections to land and spirituality, family and culture, housing, education, employment, criminal justice, and other sectors that impact health. The conditions that support an integrated systems approach to health service improvement are trained and supportive staff, strong management structures, systems and a culture of CQI, and resourcing and cost-effectiveness (25). “Because the effect of any given input depends on other conditions in a system” (21) (p. 1627), vertical and horizontal integration are not discrete processes—the two need to occur simultaneously and reinforce one another.

## Vertical Integration of CQI to Community and Broader Jurisdictions

There are considerable disparities between Australian jurisdictions in support for CQI approaches in Indigenous primary health care (11, 18, 26). Ideally, CQI action and engagement occur within and between health service, regional and national levels of the health system, addressing systemic barriers within local contexts (11). In the Northern Territory, consistent and sustained policy and infrastructure support for CQI have resulted in a progressive uptake of evidence-based CQI activities and a stronger improvement of health-care performance than that of other jurisdictions (26, 27). Performance improvement is attributed in large part to improved decision-making facilitated by the active and timely engagement of primary health-care teams in collation, analysis, participatory interpretation, and reporting of good quality local health care and health outcome data. In Queensland, initially strong but unsustained CQI support resulted in a rapid rise and subsequent fall in relevant CQI activities (27). Making the shift from traditional top down performance accountability approaches to supporting more locally driven participatory approaches has been challenging for many bureaucratic structures at Federal and State levels. In most jurisdictions, CQI initiatives have relied on local service managers and clinicians, with quality improvement efforts hampered by the poor availability of localized and timely health-care performance data (11). However, there has been substantial developmental work over recent years, and a national CQI framework for Indigenous Australian primary health care (2015–2025) is in a final consultation stage. Its imminent release and implementation should focus attention on how consistent policy and infrastructure support can be sustained to enable the wide-scale uptake of CQI activities (27).

For community-based services, engaging Indigenous community members in CQI processes is critically important for identifying and making sound decisions about priority areas for meaningful local health care and health improvement (11). Spurling (28) found that Inala (Brisbane) community members: “articulated an authoritative understanding of how interrelated, inter-generational, social, cultural, and environmental determinants of health operated in a cycle to influence the community’s health.” Further, their experience of the SCDOH varied significantly according to their age and sex, suggesting opportunities for demographically targeted policy intervention. The benefits of engagement for community members include opportunities for articulating what is important to them about health, increased sense of wellbeing and ownership over services and personal health, receiving improved health care that meets individual and community needs, and perceived social support (29). For primary health-care services, community engagement in planning, implementation, and monitoring can result in delivery of more accessible and responsive health services, improved efficiencies in business operations, and improved integration of services (30). The benefits of community participation processes have been documented in the areas of maternal and child health (31), mental health services (32), and communicable diseases (33). Best practice models recommend such “bottom up,” community-owned and led approaches (34) that focus on outcomes, with a common purpose to improve the connections between people and services (35).

## Horizontal Integration of CQI to Improve Clinical Care and the Social and Cultural Determinants of Health

Continuous quality improvement processes provide an iterative, interactive, and systemic method for extending improvement processes beyond clinical service delivery to address the social and cultural determinants of health (36). The conditions in which people are born, grow, work, live, and age play a much more important role in health than health care, as do the cultural norms and values shaping the conditions of daily life—that is, the social and cultural determinants of health (37). Between one-third and one-half of the health gap between Indigenous and non-Indigenous Australians is estimated to be associated with differences in socioeconomic position (38). Also important are health behaviors, physical environments, education, food security, community infrastructure, resources and capacities, environmental stewardship, cultural continuity and the historical and contemporary effects of colonization, racism, social exclusion, loss of land, and the stolen generations (39). Researchers are just beginning to articulate the interconnecting mechanisms and contexts through which the social and cultural determinants affect health (40). Dealing with these determinants in evolving complex environments requires dynamic improvement methods that aim to alleviate health and social inequalities, oppression, poverty, and other injustices (23).

Primary health care services are well placed to support the application of comprehensive CQI approaches both to and beyond the clinical, and there has been work on developing indicators that account for the social and cultural determinants of health in Indigenous communities (36, 41, 42). Primary health-care services have trialed CQI processes to address issues such as health promotion (43, 44) and food security in remote communities (45). CQI has been promoted as an approach to improving remote community housing and to enhancing the social determinants of child health in remote community environments of the Northern Territory (36, 46). These studies have found a wide range of physical and social determinants that underpin poor child health and developed tools for use in comprehensive primary health-care services to assess community-level social determinants of health such as water and food supply, and household-level social and environmental determinants that place children at greater risk of poor health and development outcomes. There is also evidence that CQI approaches have some potential to improve child protection processes for children and families (47) and developmental work on how CQI approaches can be applied in education [e.g., Ref. (48, 49)].

However, while the evidence-based medicine movement worked for more than 20 years to develop and apply evidence of what works in clinical health-care improvement through systematic reviews and guidelines; such systematic approaches to developing a strong evidence base for practice are less evident in other sectors. There are limitations around the availability of data and a relatively poor understanding of what constitutes good quality data. For example, state and territory education departments acknowledge that it is their responsibility to support the mental health and wellbeing of all students in an inclusive learning environment (50). Yet there is little data available to guide their efforts as to which students might need enhanced support [e.g., Ref. (51)], how such support might be provided, or how linkages with primary health-care services might best be facilitated. Further, research is thus needed to determine how CQI can support best practice more generally across sectors.

## Conclusion

In Australia, many of the conditions are in place for use of CQI to improve comprehensive Indigenous primary health care. In some Australian states and territories, there are supportive policies and opportunities in health-care reform; a CQI workforce, technical support, and data infrastructure; and activity at local health center level. Extant efforts to improve the quality of clinical care can be enhanced by extending policy support and resourcing for integrating CQI vertically in linkages between primary health-care services with governments and community members and horizontally by linking and advocating with other sectors to improve the social and cultural determinants of Indigenous health. The national CQI framework for Indigenous Australian primary health care provides an opportunity for examining the potential intervention points to improve the effectiveness and efficiency of current government investments in health.

Continuous quality improvement approaches in comprehensive primary health care offer decision-making tools and feedback loops that can respond to the complex dynamic relationships between the historical, socio-cultural, economic, and environmental elements of systems that give rise to people’s opportunities and challenges in life. Using a systems approach, we can examine, develop understanding, and intervene to change the dynamic interrelations between the various components of the PHS and broader systems that impact client health at multiple levels, and how they work together as a whole. For example, PHS can augment their health assessments to incorporate questions that are systematically asked about clients’ social and cultural determinants of health, psychosocial stressors, and community participation. PHS can also audit their current systems and processes for advocacy, partnership, and/or inter-sectoral service integration to address the SCDOH, identifying strengths, gaps, and opportunities for further refinements to better address the critical SCDOH identified through the client assessments. Further, development and evaluation of such systems approaches to CQI will enhance the potential to improve quality and integration of services and improve health outcomes.

## Author Contributions

JM, RBailie, RBainbridge, KM-B, NP, and KT made substantial contributions to the general conception of the paper; RF and DA conceptualized the application of CQI to the social determinants of health in particular Indigenous primary health-care services. JM drafted the manuscript; all other authors revised the draft manuscript critically for intellectual content. All authors provided final approval of the version to be published and agreed to be accountable for all aspects of the work in ensuring that questions related to the accuracy or integrity of any part of the work are appropriately investigated and resolved.

## Conflict of Interest Statement

The authors declare that the research was conducted in the absence of any commercial or financial relationships that could be construed as a potential conflict of interest. The reviewer CM and the handling Editor declared their shared affiliation.
